# Prescription trend and lactic acidosis in patients prescribed metformin before and after the revision of package insert for allowing metformin administration to patients with moderately decreased kidney function based on real-world data from MID-NET^®^ in Japan

**DOI:** 10.3389/fmed.2023.1294696

**Published:** 2024-01-24

**Authors:** Takashi Waki, Yusuke Okada, Yuki Kinoshita, Kazuhiro Kajiyama, Chieko Ishiguro, Yuki Nakazato, Ryota Kimura, Harumi Maniwa, Naoya Horiuchi, Toyotaka Iguchi, Yoshiaki Uyama

**Affiliations:** ^1^Office of Medical Informatics and Epidemiology, Pharmaceuticals and Medical Devices Agency, Tokyo, Japan; ^2^Office of Pharmacovigilance I, Pharmaceuticals and Medical Devices Agency, Tokyo, Japan; ^3^Office of Pharmacovigilance II, Pharmaceuticals and Medical Devices Agency, Tokyo, Japan

**Keywords:** metformin, lactic acidosis, drug prescription, safety action, pharmacoepidemiology

## Abstract

**Introduction:**

This study was conducted to understand the impact of package insert (PI) revision in Japan on 18 June 2019 to allow metformin use for patients with moderately decreased kidney function (30 ≤ estimated glomerular filtration rate (eGFR) < 60 mL/min/1.73 m^2^).

**Methods:**

A new user cohort design was employed to examine the prescription trend and the occurrence of lactic acidosis in patients prescribed metformin before and after PI revision using the Medical Information Database Network (MID-NET^®^).

**Results:**

From 12 May 2016 to 31 March 2020, 5,874 patients (before, *n* = 4,702; after, *n* = 1,172) were identified as new metformin users, including 1,145 patients (before, *n* = 914; after, *n* = 231) with moderately decreased kidney function. Although no marked changes in metformin prescription were observed before and after PI revision, the daily metformin dose at the first prescription decreased after PI revision. For both before and after PI revision, less than 10 cases of lactic acidosis occurred in all patients prescribed metformin, and no lactic acidosis was observed in patients with moderately decreased kidney function.

**Conclusion:**

The results of this study are useful for understanding the safety of metformin use in patients with decreased kidney function and suggest no worse impacts of PI revision in Japan, indicating no further safety concerns on metformin use in patients with moderately decreased kidney function under the situation with careful use and safety monitoring of metformin.

## Introduction

Lactic acidosis is well known as one of the adverse reactions during the administration of metformin (1, 2). Previously, metformin was contraindicated in the USA, EU, and Japan for patients with moderately or severely decreased kidney function (3, 4), owing to the augmented risk of lactic acidosis depending on kidney function (5) and increased blood concentration of metformin caused by delayed excretion (6).

In 2016, based on accumulated scientific evidence, the package insert (PI) of metformin was revised to limit the contraindication to patients with severely decreased kidney function whose estimated glomerular filtration rate (eGFR) was <30 mL/min/1.73 m^2^ in the USA and EU (4, 7). A similar revision in the PI of metformin was implemented on 18 June 2019 in Japan (3). The revised PI in Japan also states that the safety of metformin should be carefully monitored by initiating at a low dose and by frequent monitoring of eGFR, based on a possibility such as higher blood concentration of metformin in the Japanese population than in the non-Japanese population (8). In these conditions, metformin use is allowed in patients with moderately decreased kidney function (30 ≤ eGFR <60 mL/min/1.73 m^2^).

After PI revision in Japan, metformin is expected to be used in patients with moderately decreased kidney function (30 ≤ eGFR <60 mL/min/1.73 m^2^), but the actual impacts of PI revision have not been evaluated. Therefore, the PMDA decided to conduct a pharmacoepidemiological study to understand the impacts of PI revision on the prescription trend and the occurrence of lactic acidosis in patients prescribed metformin before and after PI revision in Japan.

## Methods

### Database

In this study, real-world data (RWD) from MID-NET^®^, a reliable and valuable database in Japan (9, 10), were used for the analysis because MID-NET^®^ stores electronic medical records, administrative claim data, and diagnosis procedure combination (DPC) data of more than 6.05 million patients (as of December 2022) in cooperation with 10 healthcare organizations, including 23 university hospitals and regional core hospitals. In this database, data on the eGFR, blood lactic acid concentration, and blood pH, which are useful for detecting lactic acidosis and kidney function, were available for analysis. The study period spanned from 1 January 2009 to 31 March 2020.

The utilization of MID-NET^®^ for this study was approved on 19 February 2020 through a discussion with the expert committee of MID-NET^®^ (11), and the actual data extraction from MID-NET^®^ for the analysis was performed on the week of 26 April 2021.

### Study design and cohort

#### Source cohort

A new user cohort design was employed to evaluate the number of patients prescribed metformin and the occurrence of lactic acidosis (see Supplementary Figure S1 for details of the study design). The source cohort comprised patients who were newly prescribed metformin [i.e., A10BA (metformin) or A10BD (combination of metformin and pioglitazone) of the anatomical therapeutic chemical (ATC) classification (World Health Organization)] or dipeptidyl peptidase IV (DPP-4) inhibitors [A10BD (combinations of alogliptin and pioglitazone, linagliptin and empagliflozin, sitagliptin and ipragliflozin, and teneligliptin and canagliflozin) or A10BH (alogliptin, anagliptin, linagliptin, omarigliptin, saxagliptin, sitagliptin, teneligliptin, trelagliptin, and vildagliptin) of the ATC code (World Health Organization)] (12) from 12 May 2016 to 31 March 2020.

Patients prescribed those drugs on and after 12 May 2016 were only selected to eliminate the effects of the recommendation by the Japan Diabetes Society on 12 May 2016, which described the contraindicated use of metformin in patients with severely decreased kidney function (eGFR <30 mL/min/1.73 m^2^) and the need for careful administration of metformin to patients with decreased kidney function in the eGFR range of 30–60 mL/min/1.73 m^2^ (13). During the identification of patients in the source cohort, the following patients were excluded: (1) patients prescribed metformin or DPP-4 inhibitors before 12 May 2016; (2) patients concomitantly prescribed both metformin and DPP-4 inhibitors at t_0_ (the first prescription date of metformin or DPP-4 inhibitors from 12 May 2016 to 31 March 2020), or patients with the first medical records (electronic medical records, administrative claim data, or DPC data) within 180 days before t_0_; and (3) patients prescribed metformin or DPP-4 inhibitors within 180 days before t_0_.

#### Primary and reference cohorts

To create the primary cohort for analysis, the following patients were excluded from the source cohort: (a) patients with records of a lactic acid concentration of ≥4 mmol/L (36 mg/dL) within 180 days before t_0_ and (b) patients without any medical record after t_0_. In the primary cohort, the patients prescribed metformin were divided into two groups based on t_0_ before and after PI revision on 18 June 2019. To select patients with moderately decreased kidney function at baseline, eGFR calculated on the day closest to t_0_ within 180 days before and at t_0_ were used. The eGFR was calculated based on the following formula: 194 × serum creatinine^−1.094^ × age^−0.287^ (×0.739 for women) (mL/min/1.73 m^2^). For analysis, kidney function was categorized as normal or mild for eGFR ≥60 mL/min/1.73 m^2^, moderate for 30 ≤ eGFR <60 mL/min/1.73 m^2^, and severe for eGFR <30 mL/min/1.73 m^2^. If the eGFR was unavailable within 180 days before or at t_0_, the kidney function of such patients was categorized as “unknown.”

Similarly, patients with moderately decreased kidney function who were newly prescribed DPP-4 inhibitors for the treatment of type 2 diabetes after 18 June 2019 [the date of the PI revision (3)] were enrolled to create the reference cohort because DPP-4 inhibitors were reported as the most prescribed medication for type 2 diabetes in Japan (14). This reference cohort was used as a reference in the analysis for the appropriate interpretation of the results in patients prescribed metformin (see Supplementary Figure S2).

The follow-up period of the cohort started 1 day after t_0_ and ended at an earlier date according to the following: (1) end date of the treatment period, (2) date of another drug prescription (metformin or DPP-4 inhibitors), (3) date of the last medical record entry within the study period, (4) day before the date of the PI revision (18 June 2019), which was only applied for patients whose t_0_ was before 18 June 2019, or (5) date of outcome occurrence. The treatment period comprised the start date and duration of the prescription with a 30-day gap and a 30-day grace period. Thus, two prescriptions for the same drug were considered continuous if the later prescription date was within 30 days of the former prescription date.

### Outcome definition

In this study, the definition of lactic acidosis was based on the clinical evaluation guideline in Japan (15). It was defined as a case in which the following criteria were met on the same day during the follow-up period: (1) lactic acid concentration ≥ 5 mmol/L (45 mg/dL) and (2) blood pH < 7.35. Hyperlactacidemia, which was used in the sensitivity analysis, was defined as ≥4 mmol/L (36 mg/dL) of lactic acid concentration.

The prescription trend was evaluated by counting the number of patients prescribed metformin per month before and after PI revision and calculating the percentage of patients prescribed metformin with moderately decreased kidney function among all patients in each month. Furthermore, the number and percentage of patients who had a daily metformin dose of ≤500 mg at the first prescription were calculated before and after PI revision.

### Statistical analysis

In the primary cohort, the number of patients who were newly prescribed metformin per month was counted. The percentage of patients with moderately decreased kidney function (30 ≤ eGFR <60 mL/min/1.73 m^2^) was also calculated every month.

Patient background such as eGFR (within 180 days before or at t_0_), age and sex (at t_0_), concomitant medication (within 180 days before t_0_; diuretics and antidiabetic drugs other than metformin and DPP-4 inhibitors), and comorbidities (within 180 days before t_0_) such as heart diseases (e.g., heart failure, angina pectoris, arrhythmia, and cardiac infarction), diabetic complications (e.g., diabetic neuropathy, diabetic kidney disease, and diabetic eye disease), and pulmonary diseases (e.g., chronic obstructive pulmonary disease and bronchitis) were tabulated for all patients, regardless of their kidney function, and for patients with moderately decreased kidney function prescribed metformin or DPP-4 inhibitors, separately. When a patient had two or more concomitant medications or comorbidities, the number of patients was separately counted for each item, resulting in a duplicate count of a patient.

Among metformin-prescribed patients with moderately decreased kidney function, the prescribed daily dose of metformin at the first prescription before and after PI revision was evaluated in patients with eGFR of 45 ≤ eGFR <60 mL/min/1.73 m^2^ and patients with eGFR of 30 ≤ eGFR <45 mL/min/1.73 m^2^, separately.

In the primary cohort, the occurrence of lactic acidosis was counted before and after PI revision in all patients prescribed metformin, regardless of their kidney function, and patients prescribed metformin with moderately decreased kidney function (30 ≤ eGFR <60 mL/min/1.73 m^2^). Then, the adjusted incidence rate ratio (aIRR) in these patients was calculated after adjusting for sex and age using Poisson regression to compare the occurrence of lactic acidosis before and after PI revision. A similar sensitivity analysis was conducted for hyperlactacidemia.

SAS version 9.4 (SAS Institute, Cary, NC, USA) was used for all analyses. In presenting data with <10 patients, such data were shown as an aggregated value considering privacy based on the MID-NET^®^ publication rule (Section 13–3) (16).

## Results

### Cohort

In total, 29,244 patients were identified as new users of metformin or DPP-4 inhibitors in the source cohort. Of these patients, 5,874 patients prescribed metformin, including 4,702 patients before and 1,172 patients after PI revision, were included in the primary cohort. Among metformin-prescribed patients with moderately decreased kidney function, 914 and 231 patients (1,145 patients in total) were identified before and after PI revision, respectively. In the reference cohort, 1,268 patients were identified as the new users of DPP-4 inhibitors with moderately decreased kidney function after PI revision (see Supplementary Figure S2 for more details).

### Patient characteristics

Table 1 shows the background of patients prescribed metformin or DPP-4 inhibitors. The population with metformin prescription in the primary cohort mainly comprised patients with normal or mildly decreased kidney function (eGFR ≥60 mL/min/1.73 m^2^) and those aged ≤65 years. Among these patients, there were slightly more number of male than female ones. These patients were frequently coprescribed with insulin or diuretics and usually had comorbidities such as heart diseases or diabetic complications. No differences were observed between patients prescribed metformin before and after PI revision. More patients prescribed metformin with moderately decreased kidney function were more than 65 years old or had comorbidities of heart disease relative to all patients. However, no major differences were found among patients prescribed metformin before and after PI revision.

**Table 1:** Background of patients prescribed metformin (primary cohort) or DPP-4 inhibitors (reference cohort).

	Metformin								DPP-4 inhibitors	
	All patients				Patients with moderately decreased kidney function (30 ≤ eGFR <60)					
	Before		After		Before		After		After	
	*n*	%	*n*	%	*n*	%	*n*	%	*n*	%
	4,702	100.0	1,172	100.0	914	100.0	231	100.0	1,268	100.0
Kidney function*										
Normal/mild, eGFR ≥ 60	3,479	74.0	896	76.5	–	–	–	–	–	–
Moderate, 45 ≤ eGFR < 60	716	15.2	182	15.5	716	78.3	182	78.8	783	61.8
Moderate, 30 ≤ eGFR < 45	198	4.2	49	4.2	198	21.7	49	21.2	485	38.2
Severe, eGFR <30	42	0.9	<10	<0.9	–	–	–	–	–	–
Unknown	267	5.7	<40	<3.5	–	–	–	–	–	–
Age**										
<65	2,508	53.3	616	52.6	240	26.3	62	26.8	133	10.5
65 ≤ <75	1,348	28.7	338	28.8	344	37.6	87	37.7	361	28.5
75 ≤	846	18.0	218	18.6	330	36.1	82	35.5	774	61.0
Sex**										
Female	1,951	41.5	463	39.5	384	42.0	77	33.3	458	36.1
Male	2,751	58.5	709	60.5	530	58.0	154	66.7	810	63.9
Concomitant medications***										
Insulin	1,155	24.6	293	25.0	229	25.1	68	29.4	297	23.4
Diuretics	565	12.0	130	11.1	204	22.3	35	15.2	295	23.3
SGLT2 inhibitors	268	5.7	106	9.0	56	6.1	21	9.1	46	3.6
GLP-1 receptor agonists	199	4.2	50	4.3	51	5.6	<10	<4.4	11	0.9
Sulfonylureas	212	4.5	33	2.8	50	5.5	<10	<4.4	40	3.2
α-Glucosidase inhibitors	176	3.7	38	3.2	40	4.4	<10	<4.4	43	3.4
Glinides	79	1.7	23	2.0	16	1.8	<10	<4.4	22	1.7
Thiazolidine	71	1.5	15	1.3	16	1.8	<10	<4.4	11	0.9
Buformin	<10	<0.3	0	0.0	<10	<1.1	0	0.0	0	0.0
Comorbidities***										
Heart diseases	1,831	38.9	454	38.7	502	54.9	111	48.1	763	60.2
Diabetic complications	1,026	21.8	234	20.0	220	24.1	53	22.9	178	14.0
Pulmonary diseases	574	12.2	134	11.4	130	14.2	27	11.7	185	14.6

A value of < 10 was presented as an aggregated value based on the MID-NET^®^ publication rule (Methods).

Before, the period before PI revision in June 2019; After, the period after PI revision in June 2019.

* 180 days before and at t_0_; ** t_0_; *** 180 days before t_0_.

### Number of patients prescribed metformin before and after PI revision

**Figure 1:**
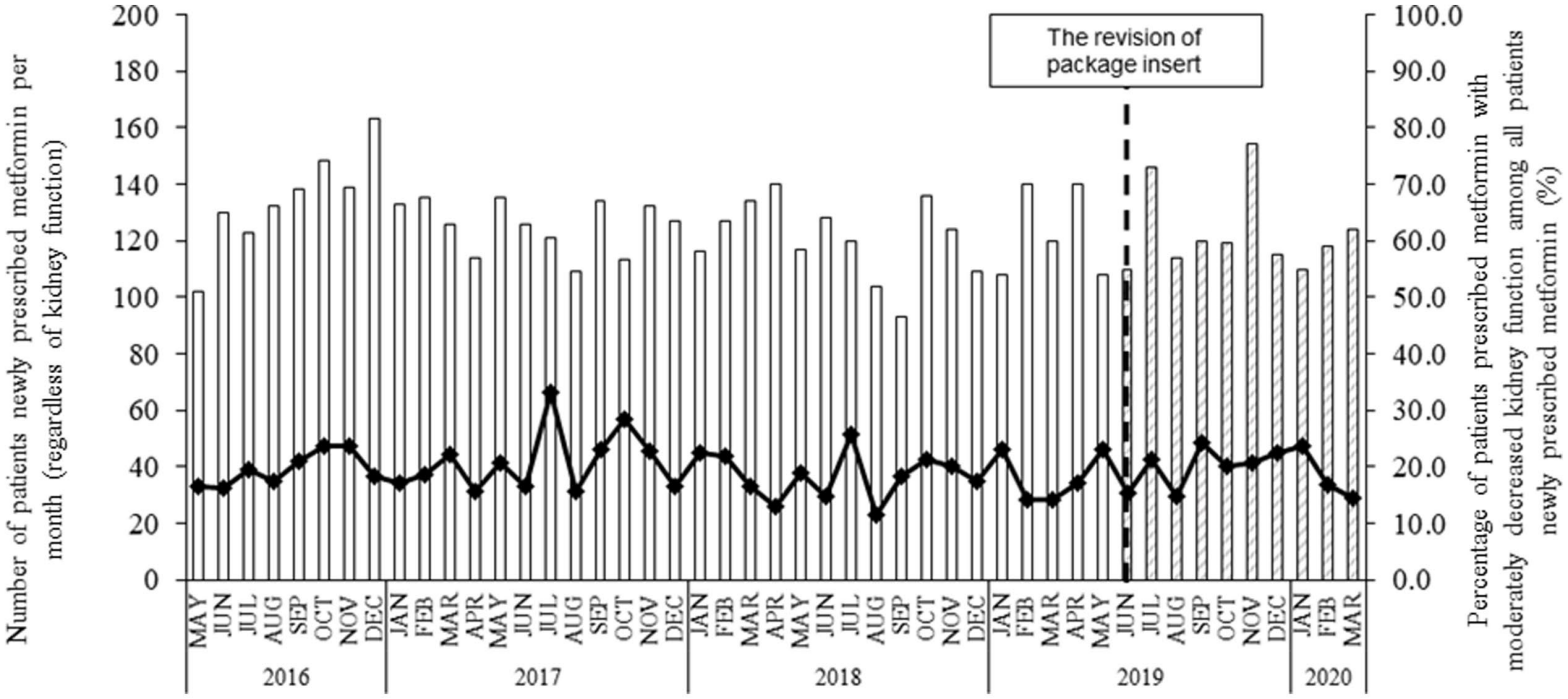
The number of patients newly prescribed metformin and the percentage of patients with moderately decreased kidney function before and after the PI revision in June 2019. Each bar indicates the number of patients prescribed metformin per month, regardless of their kidney function since 12 May 2016. “Blank” and “shaded” bars present the patient number before and after the PI revision in June 2019, respectively. The symbol with the line indicates the percentage of patients with moderately decreased kidney function among all patients prescribed metformin.

Figure 1 shows the number of patients newly prescribed metformin before and after PI revision in June 2019. Metformin was prescribed to approximately 100–150 patients per month, and 10–30% of these patients had moderately decreased kidney function. No marked changes in the number of patients or the percentages were found before and after PI revision.

### Metformin dose before and after PI revision

Interestingly, at the first prescription, the percentage of daily metformin dose ≤500 mg increased from 65.2% before PI revision to 75.5% after PI revision in patients with eGFR of 30 ≤ eGFR <45 mL/min/1.73m^2^ (Table 2A) and from 63.8% before PI revision to 68.7% after PI revision in patients with eGFR of 45 ≤ eGFR <60 mL/min/1.73 m^2^ (Table 2B).

**Table 2:** Daily metformin dose at the first prescription in patients with moderately decreased kidney function.

	(A) 30 ≤ eGFR < 45 mL/min/1.73 m^2^				(B) 45 ≤ eGFR <60 mL/min/1.73 m^2^			
	Before		After		Before		After	
	n	%	n	%	n	%	n	%
	198	100.0	49	100.0	716	100.0	182	100.0
Daily metformin dose								
≤500 mg	129	65.2	37	75.5	457	63.8	125	68.7
>500 mg	69	34.8	12	24.5	259	36.2	57	31.3

Before, the period before the PI revision in June 2019; After, the period after the PI revision in June 2019.

### Occurrence of lactic acidosis and hyperlactacidemia before and after PI revision and comparison with DPP-4 inhibitors

In all patients prescribed metformin, regardless of their kidney function in the primary cohort, fewer than 10 cases (as an aggregated value, see the “Methods” section) of lactic acidosis occurred both before and after PI revision, and the aIRR was 4.85 with a markedly large confidence interval (CI) because of the limited occurrence of lactic acidosis (95% CI, 0.44–53.63) (Table 3A). In the patients prescribed metformin with moderately decreased kidney function (30 ≤ eGFR <60 mL/min/1.73 m^2^) in the primary cohort, no cases of lactic acidosis were observed both before and after PI revision (Table 3B). In addition, fewer than 10 cases were identified in patients prescribed DPP-4 inhibitors with moderately decreased kidney function (30 ≤ eGFR <60 mL/min/1.73 m^2^) after PI revision in the reference cohort (Table 3C). In the sensitivity analysis of hyperlactacidemia, the aIRR was 2.38 with a large CI (95% CI, 0.27–21.29) compared with those before and after PI revision in all patients prescribed metformin, regardless of their kidney function in the primary cohort. No occurrence of hyperlactacidemia was observed after PI revision in patients prescribed metformin with moderately decreased kidney function (30 ≤ eGFR <60 mL/min/1.73 m^2^) in the primary cohort (data not shown).

**Table 3:** Risk of lactic acidosis in patients prescribed metformin or DPP-4 inhibitors.

	Number of patients	Total patient-years	Number of patients with lactic acidosis	Adjusted incidence rate ratio (aIRR)* (95% CI)
(A) All patients prescribed *metformin*, regardless of their kidney function				
*Before* PI revision	4,702	2,981.48	<10^#^	1.00 (reference)
*After* PI revision	1,172	299.52	<10^#^	4.85 (0.44–53.63)
(B) Patients prescribed *metformin* with moderately decreased kidney function (30 ≤ eGFR <60 mL/min/1.73 m^2^)				
*Before* PI revision	914	539.52	0	1.00 (reference)
*After* PI revision	231	55.17	0	Incalculable
(C) Patients prescribed DPP-4 inhibitors with moderately decreased kidney function (30 ≤ eGFR < 60 mL/min/1.73 m^2^)				
*After* PI revision	1,268	259.69	<10^#^	–

95% CI, 95% confidence interval.

* The incident rate ratio was calculated after adjusting for sex and age.

^#^ A value of < 10 was presented as an aggregated value based on the MID-NET^®^ publication rule (Methods).

## Discussion

In this study, the impacts of PI revision on prescription trends and the occurrence of lactic acidosis in patients prescribed metformin before and after PI revision were examined using RWD from MID-NET^®^. No changes in the number of patients prescribed metformin and the percentage of patients with moderately decreased kidney function in the primary cohort were observed before and after PI revision, indicating that PI revision has no impacts on prescription trends in patients with metformin in Japan. In fact, in the period before PI revision in Japan, a certain number of metformin prescriptions in patients with moderately decreased kidney function were identified in this study, which may be due to the potential effect of the 2016 PI revision in the USA and/or EU (4, 7), as well as the 2016 recommendation by the Japan Diabetes Society, which was basically based on the 2016 PI revision in the USA (13) because the 2016 PI revision in the USA and/or EU increased the number of metformin prescriptions to patients, which could bring to a similar situation in Japan (17, 18).

However, the increased percentage of patients who were prescribed a daily metformin dose of ≤500 mg as the first prescription after PI revision in the primary cohort suggests an effect of PI revision for appropriate selection of patients for the treatment with metformin and a more careful use of metformin by initiating at lower dose.

Regarding lactic acidosis in the primary cohort, compared with before PI revision, a higher point estimate was observed after PI revision in all patients prescribed metformin, regardless of their kidney function. However, this higher value may not necessarily indicate the increased occurrence after PI revision as the CI was markedly large owing to the limited number of occurrences of lactic acidosis (95% CI, 0.44–53.63). In fact, lactic acidosis was not observed in patients with moderately decreased kidney function (30 ≤ eGFR <60 mL/min/1.73 m^2^), despite the expected higher occurrence in this group (5). In addition, lactic acidosis occurred in the patients prescribed DPP-4 inhibitors after PI revision but not in patients prescribed metformin with moderately decreased kidney function (30 ≤ eGFR <60 mL/min/1.73m^2^) before and after PI revision. Previous studies have also reported that metformin use with dose adjustment based on renal function was safe without hyperlactatemia in patients with moderately or severely decreased kidney function (1), the occurrence of lactic acidosis between patients with type 2 diabetes using metformin and other antidiabetic drugs in Japan was not significantly different (19), and the occurrence of lactic acidosis in patients with an eGFR of 30–60 mL/min/1.73 m^2^ was not associated with metformin (20). Accordingly, the observed lactic acidosis in patients prescribed metformin in this study would not cause significant safety concerns when continuing the current safety measures such as careful use and safety monitoring of metformin.

A strength of this study was the utilization of the laboratory test results of lactic acid concentration and blood pH as the outcome of lactic acidosis from MID-NET^®^ (9, 10). However, the following limitations should be considered when evaluating the study results. First, the target population in this study may have been smaller than that in clinical practice because data from clinics were not included in MID-NET^®^ (10). Second, the results may be affected by other potential confounders such as dehydration, sick day, and lifestyle factors (e.g., alcohol consumption), other comorbidities (e.g., myocardial infarction, sepsis, and excessive bleeding), and other concomitant medications that were not included in this study. Third, the study period after PI revision (approximately 6 months) may not be sufficient relative to the period before PI revision (approximately 3 years), which would lead to the absence or limited occurrence of lactic acidosis in this study, although the study objective was to examine changes in the period early after PI revision. Further studies may be necessary to evaluate the occurrence of lactic acidosis caused by metformin over a longer period.

The PMDA conducted a safety assessment on metformin-related lactic acidosis based on the results of this study and other information, including case reports and related literature, and concluded that PI revision had no worse impacts on the occurrence of lactic acidosis in patients prescribed metformin with moderately decreased kidney function. Careful use and safety monitoring of metformin should be continued in clinical practice to manage the risk.

In conclusion, the results of this study suggest no further safety concerns on metformin use in patients with moderately decreased kidney function (30 ≤ eGFR <60 mL/min/1.73 m^2^) under the situation with careful use and safety monitoring of metformin after PI revision in Japan.

## Data availability statement

The original contributions presented in the study are included in the article/Supplementary material, further inquiries can be directed to the corresponding author.

## Ethics statement

Ethical approval was not required for the studies involving humans because, since this study was conducted as an official activity of the PMDA under the Pharmaceuticals and Medical Devices Agency Law (Article 15–5–(c) and (f)) (Pharmaceuticals and Medical Devices Agency), it was not subject to review by institutional review boards (Pharmaceuticals and Medical Devices Agency) (21, 22). The studies were conducted in accordance with the local legislation and institutional requirements. The human samples used in this study were acquired from a by-product of routine care or industry. Written informed consent to participate in this study was not required from the participants or the participants’ legal guardians/next of kin in accordance with the national legislation and the institutional requirements.

## Author contributions

TW: Conceptualization, Methodology, Writing – original draft, Writing – review & editing, Formal analysis, Investigation. YO: Conceptualization, Investigation, Methodology, Writing – original draft, Writing – review & editing. YK: Conceptualization, Formal analysis, Investigation, Methodology, Writing – original draft, Writing – review & editing. KK: Conceptualization, Methodology, Project administration, Writing – original draft, Writing – review & editing. CI: Conceptualization, Methodology, Writing – review & editing. YN: Writing – original draft, Writing – review & editing. RK: Conceptualization, Methodology, Writing – original draft, Writing – review & editing. HM: Writing – original draft, Writing – review & editing. NH: Methodology, Writing – original draft, Writing – review & editing. TI: Methodology, Writing – original draft, Writing – review & editing. YU: Conceptualization, Methodology, Supervision, Writing – original draft, Writing – review & editing.
