# Supplementary figures and images for: Prescription trend and lactic acidosis in patients prescribed metformin before and after the revision of package insert for allowing metformin administration to patients with moderately decreased kidney function based on real-world data from MID-NET® in Japan
Source: Front Med (Lausanne). 2024 Jan 24;10:1294696. doi: 10.3389/fmed.2023.1294696 (PMC10847222; doi:10.3389/fmed.2023.1294696)

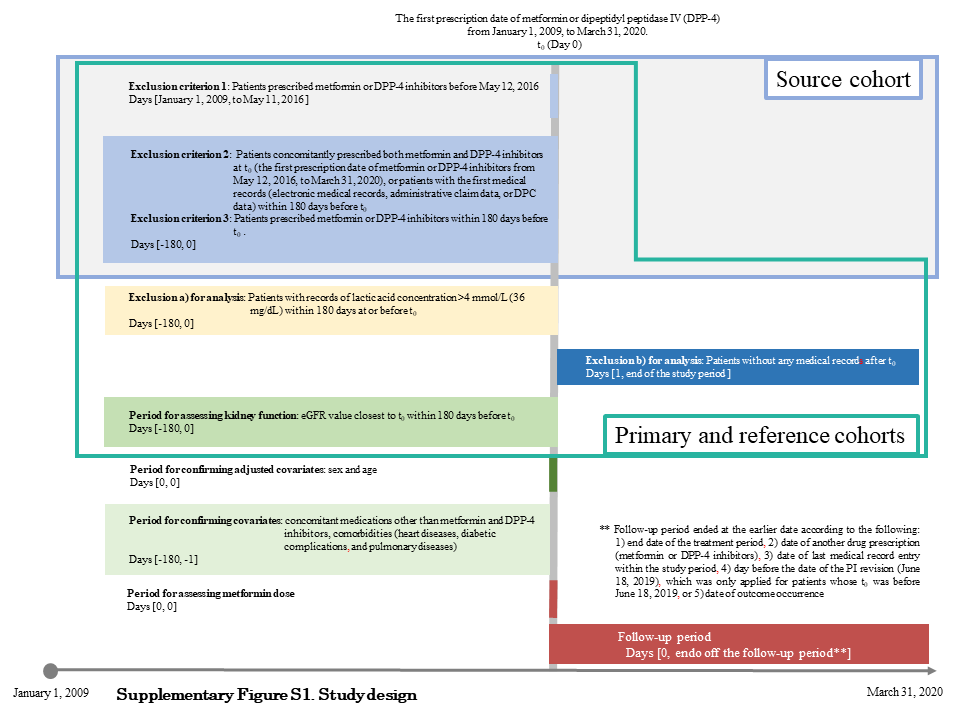

Supplement: Supplementary file 1 [file Image_1.TIF]

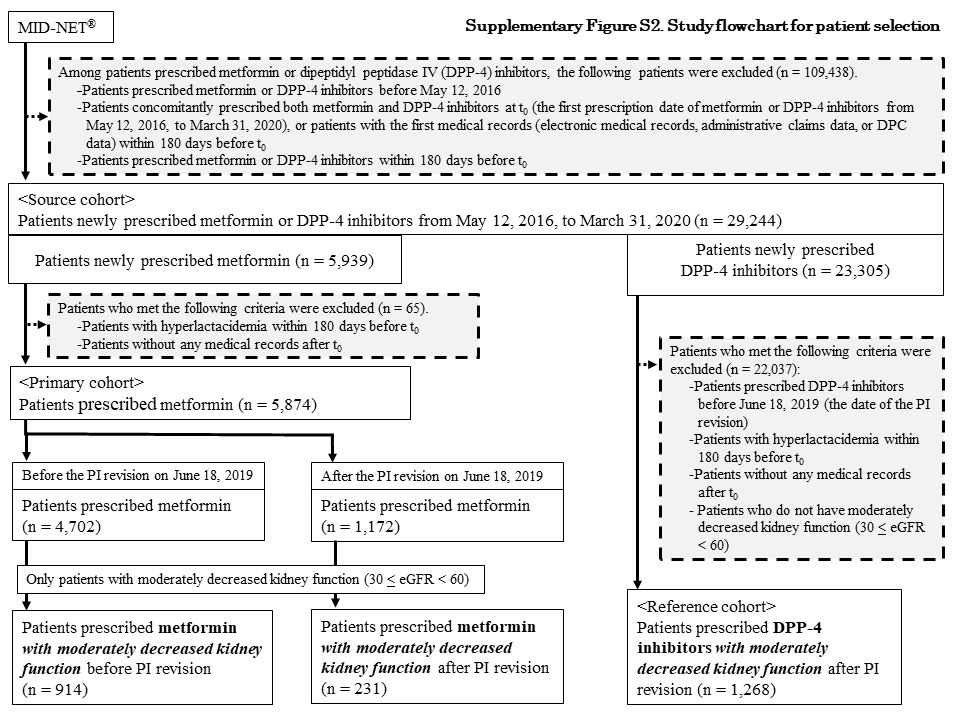

Supplement: Supplementary file 2 [file Image_2.TIF]
